# Serum miR-224 Reflects Stage of Hepatocellular Carcinoma and Predicts Survival

**DOI:** 10.1155/2015/731781

**Published:** 2015-01-22

**Authors:** Li-ping Zhuang, Zhi-qiang Meng

**Affiliations:** ^1^Department of Integrative Medicine, Fudan University Shanghai Cancer Center, 270 Dongan Road, Shanghai 200032, China; ^2^Department of Oncology, Shanghai Medical College, Fudan University, Shanghai 200032, China

## Abstract

*Background*. In our previous study, we conducted a systematic screening of miRNA to identify potential serum biomarkers for predicting venous metastasis and survival in patients with hepatocellular carcinoma (HCC). miR-224 was one of the differentially expressed miRNAs. This study aimed to confirm whether serum miR-224 level is associated with the presence of venous metastasis and survival. *Methods*. TaqMan miRNA probe was used to perform qRT-PCR assays to evaluate the expression of serum miR-224 in a cohort of 182 HCC patients. *Results*. Patients with high miR-224 serum level showed poor survival compared to that with low miR-224 serum level (HR 1.985; 95% CI, 1.08, 3.65, *P* = 0.027). The serum miR-224 levels were significantly higher in the BCLC stage C patients compared with the stage B patients (*P* = 0.005). In further analysis, significant difference of serum miR-224 expression level was observed when patients grouped by the status of PVTT but not the status of extra-liver metastasis (*P* = 0.013 and *P* = 0.091). Serum levels of miR-224 showed significant relation with parameters of liver damage and serum AFP. *Conclusion*. Serum miR-224 might be BCLC stage dependent. It can reflect the status of tumor and liver damage. It was an independent predictor for the survival of HCC patients.

## 1. Introduction 

MicroRNAs (miRNAs), single-stranded nocoding RNAs of 19–25 nucleotides, account for approximately 1% of the human genome but are assumed to regulate more than 50% of all protein-coding genes [1, 2]. The regulation of miRNAs on their target genes is mainly negative. It is reported that about half of human miRNAs localized in fragile chromosomal regions, which are associated with the onset of various human cancers [3], indicating that miRNAs are crucial in cancer development and progression. miRNAs can act as both tumor suppressors (by binding to oncogenes and suppressing them) and oncogenes (by binding to tumor suppressor genes and suppressing them) [4]. Considerable studies have shown that dysregulation of miRNAs exists in tumor tissues in comparison with adjacent nontumor tissues [5–8]. In addition, expression profiling of miRNAs has also been shown to be an accurate method of classifying cancer subtypes and predicting prognosis [9, 10]. Recent findings demonstrated that human serum and plasma contain a large amount of stable miRNAs and that the expression profile of these miRNAs holds great promise as a novel noninvasive biomarker [11].

MiR-224 is one of the most commonly overexpressed miRNAs in hepatocellular carcinoma (HCC) tissues [12]. The upregulation of miR-224 starts form the precancerous stage and persists throughout HCC development [13, 14]. The serum level of miR-224 in HCC patients has never been reported. Zhang et al. reported that miR-224 played a role in cell proliferation, migration, invasion, and antiapoptosis in HCC by directly binding to its gene targets, such as CDC42, CDH1, PAK2, BCL-2, and MAPK1 [15]. Thus, miR-224 might be a potential biomarker for predicting the aggressiveness of HCC. Venous metastasis, with tumor thrombi in the portal vein and the inferior vena cava, is a major hallmark of metastatic HCC and represents poor survival [16]. Cheng et al. reported that 40%–90.2% of advanced HCC patients had portal vein tumor thrombosis (PVTT) [17]. Even in patients with HCC tumor smaller than 2 cm, 40.5% of them had microscopic venous invasion [18]. It is an urgent need for new biomarkers to predict the development of PVTT; then precaution treatments can be taken. In our previous study, we conducted a systematic screening to identify differentially expressed miRNAs in the serum of HCC patients with different status of PVTT, which might be used as a biomarker for predicting venous metastasis and survival. The results revealed that miR-224 is one of the elevated miRNAs in the serum of HCC patients with PVTT in comparison with HCC patients without PVTT. In this study, we evaluated the expression of serum miR-224 in a larger cohort of patients to confirm whether it can be an appropriate biomarker for predicting venous metastasis and survival in HCC patients.

## 2. Materials and Methods 

### 2.1. Study Subjects

HCC patients who present in Integrative Department, Fudan University Shanghai Cancer Center, between January 2012 and July 2013, were retrospectively enrolled into the present study. Criteria for patient selection were as follows: histologically confirmed HCC or clinical diagnosis based on dynamic imaging and an underlying chronic liver disease [19]; a good performance status (ECOG level < 2). Patients with Child-Pugh stage C or a history of another malignant within the last five years were excluded. Blood samples, as well as Child-Pugh score, BCLC stage, serum AFP level, and biomarkers for liver function were obtained from each participant at the first admission to the hospital. About five milliliters of venous blood was collected in coagulation-promoting tubes. The whole blood was centrifuged at 4°C, 3000 r.p.m. for 10 min, followed by an additional centrifugation at 12,000 r.p.m. for 15 min to completely remove all remaining cells. The serum samples were portioned in aliquots and stored at −80°C until analysis. Standard parameters of liver function including total bilirubin (TBIL), direct bilirubin (DBIL), alanine aminotransferase (ALT), aspartate aminotransferase (AST), *γ*-glutamyl transferase (*γ*-GGT), lactic dehydrogenase (LDH), alkaline phosphatase (ALP), albumin (ALB), prothrombin time activity percentage (PT), international normalized ratio (INR), and alpha-fetoprotein (AFP) levels were measured at the central laboratory of the Fudan University Shanghai Cancer Center. This study was approved by the Ethics Committee of Fudan University Shanghai Cancer Center, Shanghai, China, and written informed consent was obtained from each participant, in accordance with the institutional guidelines of our hospital.

### 2.2. RNA Isolation

Small RNAs were extracted from 500 *μ*L of serum using a miR-PARIS kit (AM1556) according to the manufacturer's instructions. To allow for normalization of sample-to-sample variation in RNA isolation, synthetic Caenorhabditis elegans miRNAcel-miR-54 (purchased as a custom RNA oligo nucleotide from Qiagen) was added (50 pmol/L in a 5 *μ*L total volume) to each denatured sample.

### 2.3. Quantitative Real-Time Reverse-Transcription- (RT-) PCR Assays

We used TaqMan miRNA probes (Applied Biosystems) to perform qRT-PCR assays according to the manufacturer's instructions. Briefly, 2 *μ*L aliquot of enriched small RNAs from serum samples was reverse transcribed using the Taq-Man MicroRNA Reverse Transcription Kit (Applied Biosystems, San Diego, CA). Then 2 *μ*L of the cDNA solution was used as template for the PCR stage. No-template controls for both RT step and PCR step were included to ensure target specific amplification. All reactions were run in duplicate. The CT values of the different samples were compared using the ΔΔCT method [20].The relative expression levels of target miRNAs were normalized by cel-miR-54.

### 2.4. Statistical Methods

Baseline data were expressed as mean ± standard deviation (SD) or median values. The Kolmogorov Smirnov test was used for the test of normality on quantitative data. To analyze differences between baseline data and serum miR-224 level, Student's *t*-test analysis was performed on mean numeric data, Mann-Whitney *U* test analysis was performed on non-numeric data, and chi-squared test was used for categorical variables. The overall survival (OS) was defined as the interval between the date of a definitive diagnosis and death or to the date of the last follow-up. The Kaplan-Meier method was used to compare the OS between patients in different groups. Factors associated with the outcomes were assessed by univariate analysis and multivariate analysis using Cox regression analysis. Results were reported as hazard ratios (HR) with their 95% confidence intervals (CI). The correlation coefficients between the expression level of miRNA and laboratory parameters were calculated by using the Spearman correlation. Data were analyzed using the SPSS version 20 (IBM, Chicago, IL). All *P* values were two-tailed, and differences with *P* < 0.05 were considered statistically significant.

## 3. Results 

### 3.1. Clinicopathological Data

One hundred and eighty-two patients were retrospectively recruited into this study. The majority of patients were men and long-term hepatitis B virus (HBV) carriers, which accounted for 85.2% and 87%, respectively. 53.8% of patients were diagnosed at BCLC B stage, and another 46.2% of patients presented with BCLC C stage. All patients had preserved liver function of Child A/B. 97.3% of patients received local therapy. Approaches including radiotherapy, transcatheter arterial chemoembolization, percutaneous radiofrequency ablation, percutaneous ethanol injection, and high intensity focused ultrasound. 18.1% of patients had sorafenib treatment. Patients were grouped in subjects with low or high serum miR-224 using the median miR-224 expression level of the 182 cases as cut-off value. Patients' characteristics in these two groups were summarized in Table 1. Parameters which related to liver function (DBIL, ALB, PT, INR, Child-Pugh stage), inflammatory activity in the liver (ALT, AST, *γ*-GGT, LDH, ALP), and tumor stage (BCLC stage, AFP) were significantly different between two groups. In addition, the distributions of gender and HBV infection were also different. The results indicated that the serum miR-224 level might be affected by multiple factors.

### 3.2. Serum miR-224 Is a Prognostic Marker in HCC Patients

**Figure 1:**
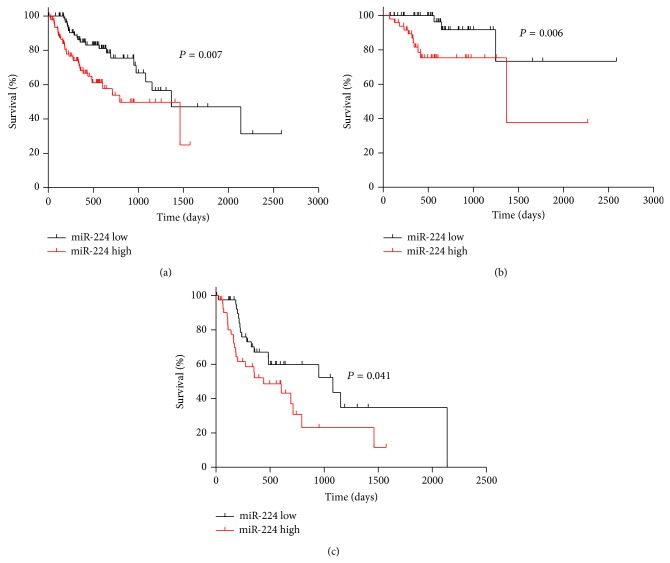
Kaplan-Meier curve for patients with low and high miR-224. (a) The median miR-224 expression level was chosen as the cut-off point for separating the miR-224 low-level cases (*n* = 91) from the miR-224 high-level cases (*n* = 91). Low level of serum miR-224 was associated with better survival. (b) In patients with BCLC B stage, low level of serum miR-224 can also predict better survival. (c) In patients with BCLC C stage, low level of serum miR-224 favors better survival.

Kaplan-Meier analysis revealed that patients with low level of serum miR-224 had favorable trends of OS (log rank = 8.222, *P* = 0.007, Figure 1(a)). The median survivals for patients with a low level of serum miR-224 and that with a high level of serum miR-224 were 623 (95% CI, 522–725) days and 437 (95% CI, 367–507) days, respectively. BCLC stage is a critical factor for HCC prognosis. We then grouped patients according the BCLC stage and compared the survival curves according to miR-224 levels in each subgroup of patients. The significant differences were evident, which indicated that high level of serum miR-224 presented poor survival (*P* = 0.006 and *P* = 0.041, resp., Figures 1(b) and 1(c)).

**Figure 2:**
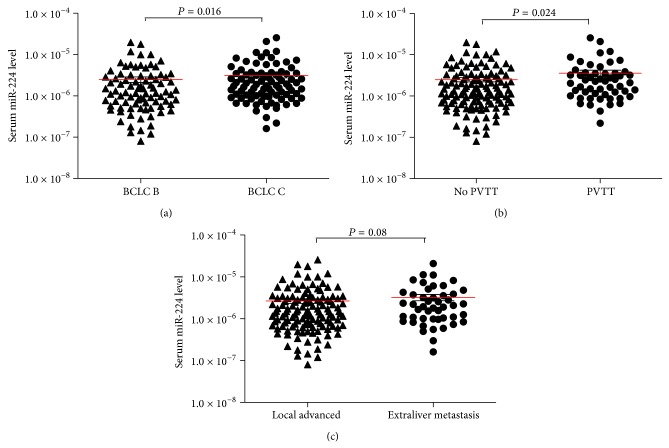
Serum miR-224 level in different subgroups of patients. (a) A comparison ofmiR-224 expression levels in HCC patients with BCLC B stage and BCLC C stage. Statistical significance was calculated. (b) Serum miR-224 in HCC patients with PVTT was significantly higher than that without PVTT. (c) No significant difference of serum miR-224 level in patients with local advanced HCC and extra-liver metastasis was evident.

To examine whether the serum miR-224 levels were associated with specific stages of the disease, the patients were classified according to the BCLC stage. Of the 182 patients with HCC, the serum miR-224 levels were significantly higher in the stage C patients compared with the stage B patients (*P* = 0.005; Figure 2(a)). In further analysis, we found that the significant difference of serum miR-224 expression level was observed when patients grouped by the status of PVTT but not the status of extra-liver metastasis (*P* = 0.013 and *P* = 0.091, Figures 2(b) and 2(c)), suggesting that the serum miR-224 level might be associated with the presence of PVTT. Additionally, we performed Cox proportional hazards regression analysis to exclude the confounder effect. A multivariate analysis confirmed that the serum miR-224 level was an independent prognostic factor for OS (HR 1.985; 95% CI, 1.08, 3.65, *P* = 0.027) (Table 2).

### 3.3. Serum Levels of miR-224 Correlate with Liver Function and Inflammatory Disease Activity in the Liver

Aforementioned, variables associated with liver function and inflammation in liver were significantly different in patients with low serum miR-224 level when compared with high serum miR-224 level. Thus, we conducted the Spearman analysis to confirm the correlation between serum miR-224 level and each liver related parameter (Table 3). The results showed that serum levels miR-224 showed significant relation with parameters of liver damage, such as ALT, AST, *γ*-GGT, LDH, and ALP. In addition, significant correlation between serum level of miR-224 and AFP was also evident.

## 4. Discussion 

HCC, a highly malignant cancer, shows the propensity of intrahepatic spreading and extrahepatic metastasis [21]. Presence of PVTT is a clinicopathological feature of metastatic HCC, which represents that HCC cells have acquired molecular changes that enable them to detach from the primary tumor mass, invade into the blood vessel, and survive in the circulatory system [22, 23]. miRNAs are reported to be highly involved in multiple cellular processes, such as proliferation, apoptosis, migration, and angiogenesis [24]. Based on the systematic screening, the expression patterns of miRNA in primary HCCs and corresponding venous metastases have ever been reported. The results were inconsistent. Study of Liu et al. showed that several differentially expressed miRNAs were found between PVTT tissues and the paired tumor tissues, and miR-135a showed the greatest increase in PVTT tissue [25]. However, Wong et al. reported that the miRNA expression profiles of primary HCCs and corresponding venous metastases were similar [26].

miRNAs in circulation are stable and easily accessed [27]. Growing evidences suggest that they are promising biomarkers for predicting the aggressiveness of cancer [28]. The concern that circulating miRNAs have a heterogeneous origin and they are not always directly associated with changes occurring in tumor tissues has limited their application. Contrarily, we think that the indirect reflection effect of circulating miRNAs might be another advantage for them to be biomarkers. Accumulating studies show the importance of microenvironment in the onset and progression of cancer, especially for HCC that usually arises from chronic liver disease [29, 30]. The formation of PVTT is reported to be linked to an immune-subversive microenvironment. Briefly, the persistent presence of HBV in the liver tissue elevated TGF-*β* activity, which suppressed miRNA-34a, leading to enhanced production of CCL22. CCL22 then recruited regulatory T cells to facilitate immune escape [31]. Circulating miRNAs which can directly reflect the changes of tumor, also can indirectly reflect the changes of microenvironment would be promising biomarkers.

PVTT in HCC patients is one of the most significant factors for the dismal outcomes [32, 33]. Biomarkers related to the development of venous metastasis are likely to provide more accurate assessment of prognosis of HCC patients. Circulating miRNA profile in HCC patients with PVTT has not yet been reported. The dysregulation of miR-224 varies in tumors derived from different organs. For example, miR-224 is upregulated in tumor tissues of cervical cancer [34] and renal cancer [35], whereas in prostate cancer [36] it is downregulated. In our study, BCLC C stage patients have higher serum miR-224 level than that in BCLC B stage patients. Higher serum miR-224 level represents poor survival, indicating that miR-224 is an oncomir in HCC, which is consistent with the expression pattern of miR-244 in HCC tissues. Subgroup analysis revealed that serum miR-224 level correlated with the status of PVTT but not the status of extra-liver metastasis, suggesting that the serum miR-224 level might be a predictor for the development of PVTT. Furthermore, serum miR-224 level was found to be significantly correlated with parameters of liver damage, which indicated that serum miR-224 also can reflect liver damage. The possible mechanisms might be that liver damage induced inflammation activated inflammatory pathways, such as p65/NF kB. And p65/NF kB is identified as a direct transcriptional regulator of miR-224 expression [37]. Liver inflammation and liver cell damage are common in HCC patients due to the history of chronic liver disease. The formation of PVTT may result in the spread of tumor cells via portal vein. In addition, portal vein obstruction also causes further deterioration in liver function. But we can exclude the possibility that miR-224 merely reflects liver damage, because significant relation is also evident between miR-224 and serum AFP, which indicates that it also can reflect the status of tumor.

In conclusion, serum miR-224 is an independent predictor for the survival of HCC patients. The expression of miR-224 might be BCLC stage dependent. It can reflect the status of tumor and liver damage.

## Figures and Tables

**Table 1:** Patients' characteristics.

Parameter	miR-224 low (*n* = 91)	miR224 high (*n* = 91)	*P* value
Age, years, mean ± SD	54 ± 11.8	52.5 ± 9.3	0.366
Gender (male/female)	69/22	86/5	0.001
Hepatitis B, *n* (%)	74 (81.3)	85 (93.4)	0.015
TBIL (umol/L)	17.1 ± 8.8	19.1 ± 12.7	0.103
DBIL (umol/L)	5.7 ± 3.4	7.9 ± 7.2	0.022
ALT (IU/L)	34.6 ± 20.9	59.4 ± 65.9	0.000
AST (IU/L)	40.2 ± 23.8	80.5 ± 81.9	0.000
*γ*-GGT (IU/L)	126.5 ± 151.7	215.5 ± 223.1	0.000
LDH (IU/L)	198.9 ± 88.9	286.7 ± 177.4	0.000
ALP (IU/L)	122.1 ± 86.2	167 ± 126.7	0.000
ALB (g/L)	40.1 ± 4.8	38.2 ± 4.6	0.007
PT (s)	12.6 ± 0.96	12.9 ± 1.04	0.011
INR	1.07 ± 0.08	1.09 ± 0.09	0.018
Child-Pugh (A/B)	87/4	76/15	0.03
BCLC (B/C)	58/33	40/51	0.012
AFP (ng/mL)	1049 ± 1442	1822 ± 1702	0.001

TBIL: total bilirubin; D-TBIL: direct bilirubin; ALT: alanine aminotransferase; AST: aspartate aminotransferase; *γ*-GGT: *γ*-glutamyl transferase; LDH: lactic dehydrogenase; ALP: alkaline phosphatase; ALB: albumin; PT: prothrombin time activity percentage; INR: international normalized ratio.

**Table 2:** Univariate and multivariate Cox regression analyses of parameters associated with overall survival of all HCC patients.

Parameters	Univariate analysis		Multivariate analysis	
	HR (95% CI)	*P* value	HR (95% CI)	*P* value
Gender (male versus female)	1.659 (0.66, 4.172)	0.282		
HBV (no versus yes)	0.767 (0.375, 1.567)	0.466		
Cirrhosis (no versus yes)	1.268 (0.506, 3.181)	0.613		
AFP (<200 versus >200 ng/mL)	2.397 (1.339, 4.289)	0.003	1.998 (1.085, 3.681)	0.026
Child-Pugh (A versus B)	2.04 (1.041, 3.996)	0.038	/	/
BCLC (B versus C)	4.083 (2.228, 7.482)	0.000	2.865 (1.500, 5.472)	0.001
Local therapy	0.235 (0.084, 0.654)	0.006	0.170 (0.065, 0.444)	0.000
Sorafenib	1.423 (0.791, 2.562)	0.239		
Serum miR-224 (low versus high)	2.188 (1.264, 3.786)	0.005	2.085 (1.142, 3.807)	0.017

**Table 3:** Correlation of serum miR-224 levels and laboratory parameters.

Variables	Rank correlation coefficient (*r*)	*P* value
TBIL (umol/L)	0.116	0.121
D-TBIL (umol/L)	0.145	0.053
ALT (IU/L)	0.297	0.000
AST (IU/L)	0.399	0.000
*γ*-GGT (IU/L)	0.302	0.000
LDH (IU/L)	0.337	0.000
ALP (IU/L)	0.222	0.003
ALB (g/L)	−0.109	0.147
AFP (ng/mL)	0.270	0.000
PT	0.093	0.212
INR	0.094	0.209
